# Model-informed drug discovery and development approaches to inform clinical trial design and regulatory decisions: A primer for the MENA region

**DOI:** 10.1016/j.jsps.2024.102207

**Published:** 2024-11-27

**Authors:** Mohammed S. Alasmari, Salwa Albusaysi, Marwa Elhefnawy, Ali M. Ali, Khalid Altigani, Mohammed Almoslem, Mohammed Alharbi, Jahad Alghamdi, Abdullah Alsultan

**Affiliations:** a Department of Pharmaceutical Services, Security Forces Hospital, Riyadh 11481, Saudi Arabia; b Department of Pharmaceutics, Faculty of Pharmacy, King Abdulaziz University, Jeddah, Saudi Arabia; c PumasAI, Baltimore, USA; d Ifakara Health Institute, Tanzania; e Department of Clinical Pharmacy, College of Pharmacy, Najran University, Saudi Arabia; f Certara Drug Development Solutions, Certara, USA; g Saudi Food and Drug Authority, Saudi Arabia; h Department of Clinical Pharmacy, College of Pharmacy, King Saud University, Riyadh 11451, Saudi Arabia

**Keywords:** Drug development, PK/PD, PBPK, MENA region, Clinical trials, Pharmacometrics, Quantitative pharmacology, Population pharmacokinetics

## Abstract

Model-Informed Drug Discovery and Development (MID3) represents a transformative approach in pharmaceutical research, integrating quantitative models to inform and optimize decision-making throughout the drug development process. This review explores the current applications, challenges, and future prospects of MID3 within the Middle East and North Africa (MENA) region. By leveraging local data and advanced computational techniques, MID3 has the potential to significantly enhance the efficiency and success rates of drug development tailored to regional health priorities. We discussed successful case studies of applying MID3 at different phases of drug development and clinical trials. Furthermore, we emphasized the critical need for MENA countries to embrace MID3 by investing in workforce training, aligning regulatory frameworks, and fostering collaborative research initiatives. This call to action underscores the importance of a robust MID3 ecosystem, urging policymakers, academic institutions, and industry stakeholders to prioritize and support its integration into the MENA region’s healthcare.

## 1 Introduction

Model-Informed Drug Discovery and Development (MID3) is a comprehensive approach that integrates quantitative models to inform decision-making throughout the drug development process (Marshall et al., 2019, 2016). The MID3 concept focuses on the development and application of mathematical models of biological, physiological, pathological, and pharmacological processes based on preclinical and clinical data, aiming to improve the quality, efficiency, and cost-effectiveness of drug development process. The MID3 is the most recent term evolved from earlier terms including Model-Informed Drug Development (MIDD), Model-Based Drug Development (MBDD), Pharmacometrics (PMx), System Pharmacology (SP), and Modeling and Simulation (M&S) (Marshall et al., 2016). In the past two decades, the utilization of MID3 approaches has increased to support regulatory and clinical decision making (Alsultan et al., 2020, Huang et al., 2013, Lavé et al., 2016, Lee et al., 2011, Milligan et al., 2013, Rowland et al., 2011, Tuntland et al., 2014, Wang et al., 2019). The increased interest by regulatory agencies and pharmaceutical companies is mainly due to the high rates of attritions in clinical development (Sun et al., 2022). One of the main reasons for this higher attrition is safety concerns and the inability to demonstrate efficacy during clinical drug development (Lee et al., 2011). MID3 approaches (e.g., population pharmacokinetics/pharmacodynamic, physiologically-based pharmacokinetics (PBPK), quantitative system pharmacology (QSP), exposure–response relationship, model based *meta*-analysis (MBMA), clinical trial simulation (CTS), disease progression models, drug-trial-disease model) can help with optimizing clinical trial design and drug dosing and supporting safety and efficacy to reduce drugs attrition (Kimko and Pinheiro, 2015, Madabushi et al., 2022, Wang et al., 2019).

As part of the strong interest in this field, several regulatory agencies have published guidelines on using modeling and simulation tools during drug development. The first guide was published by the US Food and Drug Administration (FDA) in 1999 for population pharmacokinetics studies, this was followed by European Medical Agency (EMA) guideline on population pharmacokinetics in 2007, and FDA guide for PBPK in 2007 (Table 1). In Australia, regulatory agencies started discussing the role of PK/PD modeling and simulation in 1997 (Hennig et al., 2019). Over the past 10 years, several other regulatory agencies started emphasizing the role of quantitative methods in drug development such as China, Japan and South Korea (Lee and Jeon, 2019, Li et al., 2019, Sato et al., 2017). Currently, nearly all new submissions for new drug applications (NDA) contains information related to mathematical modeling and simulation.

**Table 1:** FDA and EMA guidelines for modeling and simulation.

**Agency**	**Approach**	**The guideline**
FDA	PPK	https://www.fda.gov/regulatory-information/search-fda-guidance-documents/population-pharmacokinetics
FDA	PBPK	https://www.fda.gov/regulatory-information/search-fda-guidance-documents/physiologically-based-pharmacokinetic-analyses-format-and-content-guidance-industry
FDA	PBPK	https://www.fda.gov/regulatory-information/search-fda-guidance-documents/use-physiologically-based-pharmacokinetic-analyses-biopharmaceutics-applications-oral-drug-product
FDA	E-R	https://www.fda.gov/regulatory-information/search-fda-guidance-documents/exposure–response-relationships-study-design-data-analysis-and-regulatory-applications
FDA	FIH	Guidance for Industry: Estimating the Maximum Safe Starting Dose in Initial Clinical Trials for Therapeutics in Adult Healthy Volunteers https://www.fda.gov/downloads/drugs/guidances/ucm078932.pdf
EMA	PPK	https://www.ema.europa.eu/en/reporting-results-population-pharmacokinetic-analyses-scientific-guideline
EMA	PBPK	https://www.ema.europa.eu/en/reporting-physiologically-based-pharmacokinetic-pbpk-modelling-simulation-scientific-guideline
EMA	Pediatric PPK	https://www.ema.europa.eu/en/documents/scientific-guideline/guideline-role-pharmacokinetics-development-medicinal-products-paediatric-population_en.pdf
EMA	Obese PPK	https://www.ema.europa.eu/en/documents/scientific-guideline/reflection-paper-investigation-pharmacokinetics-obese-population-scientific-guideline_en.pdf
EMA	PK/PD	https://www.ema.europa.eu/en/documents/scientific-guideline/guideline-use-pharmacokinetics-and-pharmacodynamics-development-antimicrobial-medicinal-products en.pdf
EMA	PK	https://www.ema.europa.eu/en/human-regulatory-overview/research-and-development/scientific-guidelines/clinical-pharmacology-pharmacokinetics
EMA	FIH	van Gerven J, Bonelli M. Commentary on the EMA Guideline on strategies to identify and mitigate risks for first-in-human and early clinical trials with investigational medicinal products. Br J Clin Pharmacol. 2018 Jul;84(7):1401–1409. https://doi.org/10.1111/bcp.13550. Epub 2018 May 30. PMID: 29451320; PMCID: PMC6005602.

EMA: European Medical Agency, E-R: exposure-response relationship, FDA: Food and Drug Administration, FIH: First in Human, PPK: population pharmacokinetic, PBPK: physiologically based pharmacokinetic, PK/PD: pharmacokinetic/pharmacodynamic.

While several developed countries have made significant strides in incorporating modeling and simulation into their research on drug discovery and development, the Middle East and North Africa (MENA) region still has substantial room for growth in this area. Increasing the application of MID3 approaches in the MENA region is crucial for improving the efficiency of drug development which can lead to faster and more effective production of new drugs into markets. These quantitative methods are also essential for addressing health challenges unique to the region by enabling more precise and tailored treatments. Moreover, they can stimulate local pharmaceutical innovation, drive economic growth, and enhance the region’s competitiveness in the global market. This is important, as MENA population’s representation in clinical trials is low even compared to emerging markets (Ibrahim et al., 2020, Nair et al., 2013). Therefore, it is important to highlight and raise awareness of MID3 concepts in MENA region. The objective of this paper is to provide a summary on quantitative methods used in drug development to enhance the MENA pharmaceutical sector, thereby improving drug development efficiency, addressing regional health challenges more effectively, and fostering local innovation.

## 2 Historical background

The foundations of PK/PD modeling were laid in the early 20th century with the work of scientists A.J. Clark, who developed mathematical models to describe the effects of drugs (B., 1933). The concepts of concentration-effect relationships and the importance of the time course of drug concentration and the field of PK/PD modeling grew significantly during the 1960 s (Csajka and Verotta, 2006). Scientists Lewis Sheiner and Stuart Beal were instrumental in formalizing the principles of PK/PD modeling (Sheiner, 1997, Sheiner and Beal, 1980). With the advent of more powerful computers and software, the 1980 s and 1990 s saw a surge in the use of PK/PD models in drug development. Initially it started with the use of population PK/PD modeling (also known as nonlinear mixed-effects modeling) (Keizer et al., 2013). Later, in the 2000 s, there was a reemergence of PBPK models. Although the concept of PBPK modeling is old and dates back to the work of Teorell in 1937 (Paalzow, 1995, T., T., , 1937), it wasn't until the 1960 s that the first PBPK models, which considered the body as a series of interconnected compartments representing organs and tissues, began to be developed. These models became increasingly sophisticated, incorporating more complex descriptions of physiological processes and drug interactions with the body. Today, population PK/PD and PBPK modeling are the most commonly used modeling tools during drug development (Madabushi et al., 2022, Marshall et al., 2019, Wang et al., 2019). They are used to predict human pharmacokinetics from preclinical data, optimize dosing regimens, and support personalized medicine approaches. The integration of these models into the drug development pipeline has the potential to reduce the time and cost associated with bringing new therapeutics to market, while also improving their safety and efficacy profiles.

## 3 Regulatory perspectives of MID3 applications

Regulatory agencies, including FDA and EMA, require evidence that drugs are safe and effective in the population for which they are intended. Various MID3 approaches have been recognized by regulatory agencies as decision tools to support efficacy and safety of drugs. Modeling approaches, such as population PK/PD and PBPK are often mandated as part of the drug approval process for special populations (Jones et al., 2015, Shepard et al., 2015, Wagner et al., 2015). Starting in the 1990 s and continuing into the 21st century, FDA and EMA have issued guidelines that outline how modeling and simulation can be used to support drug approval processes (Table 1). This has led to an increased use of modeling and simulation in various, if not all, stages of drug development, from preclinical studies to clinical trial design and post-marketing surveillance. Jamei M (2016) compiled a list of approved drugs in which modeling and simulation played a role in informing their labels (Jamei, 2016). Also, several papers have been published summarizing the impact of modeling and simulation on drug approvals and labeling decisions by the FDA, these are summarized in Table 2 (Lee et al., 2011, Ogasawara and Alexander, 2019, Zhang et al., 2020).

**Table 2:** Summary of studies assessing the impact of modeling and simulation on drug approvals and labeling by the FDA.

**Drug submissions reviewed and year**	**Approach**	**Impact**
198 Biological License Application and New Drug Application (NDA) from 2000 to 2008	Pharmacometrics (most were population pharmacokinetics and exposure response analysis)	Pharmacometrics analysis influenced the approval and labeling for over 60 % of submissions. The impact of pharmacometrics increased over time from 40 % in 2004 to 75 % in 2008.
86 approved biologics between 2003–2017	Population pharmacokinetics	76 % of applications included population pharmacokinetics
Percentage of new drug applications (NDA) that contained PBPK analysis from 2013 to 2019	PBPK	20 % of NDA’s contained PBPK analysis in 2013, the number increased to 45 % in 2019.

For regulatory agencies in the MENA region, the Saudi Food & Drug Authority (SFDA) recently started incorporating MIDD in its evaluation process. This increased adoption of MIDD approaches by applicants submitting to the SFDA reflects the growing recognition of the value that these techniques can bring to the drug development and regulatory decision-making processes. Applications of MIDD include that for new drug applications, applications for adding new indications or extending indications to special populations. This trend is also observed in clinical trial applications, where applicants have applied MIDD to guide the advancement of their clinical development. The types of models encountered by the SFDA include population pharmacokinetics (PPK), mechanistic models such as physiologically-based pharmacokinetics (PBPK), and exposure–response (ER) relationships. The SFDA's evaluation approach for these models typically involves a matrix-based assessment, which aims to identify and manage the risks associated with using such computational models, whether as a replacement for clinical trials, for guiding dose selection, or for other purposes. The SFDA's experience in evaluating these modeling and simulation applications showcases its commitment to staying at the forefront of this evolving field and ensuring the appropriate use of MIDD to support regulatory decisions.

The international council for harmonization of technical requirements for pharmaceuticals for human use (ICH) endorsed the concept paper for general principles related to MIDD in November 2022 (The International Council for Harmonisation, 2022). This recognized that despite the increase in regulatory submissions and industry-regulatory interactions using evidence generated by MIDD, there is an absence of harmonized documentation standards and assessment expectations. The aim of the guideline is to harmonize expectations regarding planning, model evaluation, and documentation of evidence derived from MIDD. A key aspect is also to create a common understanding across scientists, both between and within industry and regulatory bodies. A draft of this guideline will be published for public comments in line with the anticipated 3-year timeline of this project.

## 4 Various MID3 approaches used in drug discovery and development

**Fig. 1:**
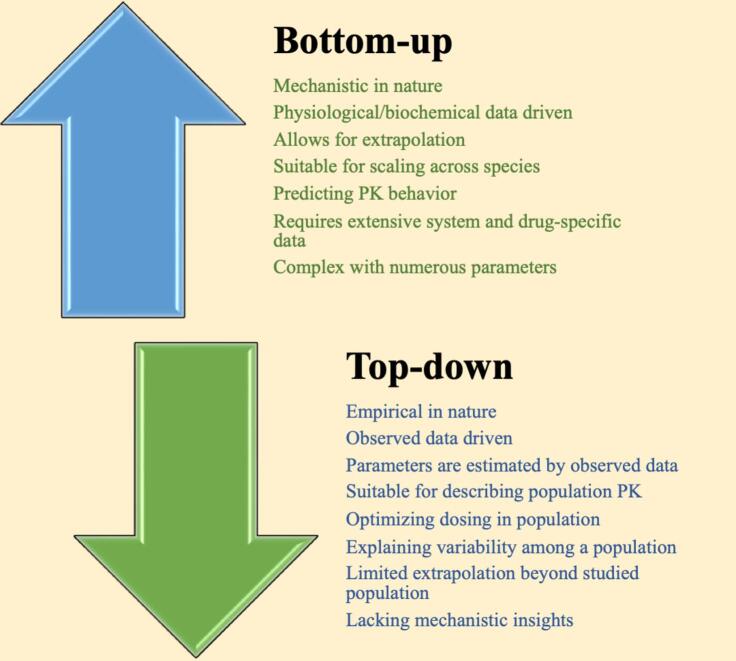
Comparison of top-down and bottom-up modeling approaches. The figure illustrates the key differences between the top-down approach which relies on empirical data from clinical studies to describe drug behavior in populations, and the bottom-up approach, which uses mechanistic models, such as PBPK, to predict pharmacokinetic based on physiological and biochemical properties. Together, these approaches offer complementary insights into drug dosing and response.

In drug discovery and development, a variety of modeling approaches are utilized to enhance the understanding and prediction of drug kinetic and dynamic behaviors. Key approaches include population pharmacokinetics/pharmacodynamic modeling which is empirical in nature, and PBPK/QSP which is mechanistic in nature (Marshall et al., 2016). The empirical model starts from data collection and taking all available pharmacokinetic information such as plasma concentration, dosing information and the time of dosing and sample collections, which is called “top-down”, while the mechanistic model starts from what is understood at the organ, or tissue level such as hepatocytes, human liver microsomes and recombinant CYP450 enzymes, which is called “bottom-up” (Fig. 1). The most widely used empirical approach method is the population pharmacokinetics/pharmacodynamic approach which is known as nonlinear mixed effect model (NLME). On other hand, the most widely used mechanistic modelling method is PBPK/QSP (Aarons, 2005, Marshall et al., 2016).

Population pharmacokinetics is a branch of pharmacokinetics that studies the variability in drug concentrations within a patient population (Ette et al., 2004, Ette and Williams, 2004a, Ette and Williams, 2004b, Vozeh et al., 1996). This approach models how drugs are absorbed, distributed, metabolized, and excreted, accounting for diverse factors that influence these processes, such as age, weight, gender, genetic factors, disease states, and concomitant medications. The key objectives of population pharmacokinetics are to quantify the typical pharmacokinetic parameters (e.g., clearance, volume of distribution) and their variability within a population, identify and understand covariates that explain the variability in drug response, and to predict drug concentrations in patients to optimize dosing regimens (Ette et al., 2004). Population pharmacokinetics employs statistical and mathematical modeling techniques to analyze data from clinical trials and real-world settings. NLME models commonly used to describe the central tendency (fixed effects) and the variability (random effects) of pharmacokinetic parameters in populations (Bauer, 2019a, Bauer, 2019b). Population pharmacokinetics is applicable in individualizing drug therapy, supporting drug development, guiding dose selection, optimizing clinical trial designs, and informing regulatory decisions and labeling by providing comprehensive pharmacokinetic insights across different subpopulations (Ette et al., 2004). Overall, population pharmacokinetics enhances our understanding of drug behavior in diverse populations, contributing to safer and more effective therapeutic interventions.

In population PK/PD modeling, pharmacokinetic behavior of drug (PK) is combined with the drug's biological effects (PD) to optimize therapeutic strategies and ensure safety and efficacy (Derendorf and Meibohm, 1999). PK/PD modeling examines the relationship between drug concentration and its therapeutic or toxic effects (known as exposure–response relationship), providing insights into the drug's efficacy and safety profile at different doses. PK/PD modeling aids in determining the optimal dosing regimen by balancing efficacy and safety, thus reducing the time and cost associated with trial-and-error approaches in clinical trials.

PBPK models integrate physiological, biochemical, and drug-specific data to simulate how drugs are absorbed, distributed, metabolized, and excreted in various populations, especially those underrepresented in clinical trials (e.g., pediatrics, elderly, renal/hepatic impairment) (Zhao et al., 2011). PBPK models can predict drug concentrations and pharmacokinetics in specific populations where clinical data is lacking, such as pediatrics, pregnant, geriatrics, and population with organs dysfunction (Lin et al., 2022). Moreover, PBPK modeling is valuable for predicting drug interactions, especially those mediated by cytochrome P450 enzymes. PBPK models help in determining necessary dose adjustments without requiring additional clinical studies, thus optimizing safety and efficacy. This capability can expedite drug development and broaden drug indications by reducing the need for extensive clinical trials in these groups. There has been a growing acceptance and use of PBPK models in regulatory submissions (Wu et al., 2023, Zhao et al., 2011). This trend is supported by regulatory guidance and ongoing collaboration between industry, academia, and regulatory agencies, aiming to improve the standardization and reliability of these models.

In contrast to empirical approach for exposure response relationship using population PK/PD modeling, QSP represents the mechanistic approach for exposure response relationship. QSP considered as a framework for understanding the complex interactions between drugs and biological systems, such in cascade biomarkers interactions, and it is a more comprehensive and predictive method for drug development (Androulakis, 2016). QSP model integrates mathematical modeling, experimental data, and systems biology to simulate the effects of drugs on biological systems at multiple scales. The key components of a QSP model, including drug pharmacokinetics, target engagement, and downstream effects, and emphasizes the importance of incorporating biological context into drug development. It is very important to integrate quantitative data from multiple sources, such as in vitro and in vivo experiments, to build a comprehensive model of drug action.

## 5 Software and tools supporting the MID3

A variety of software and tools are available to facilitate PK and PD analyses, each with its own strengths and applications. For population PK/PD modeling, NONMEM (Nonlinear Mixed Effects Modeling) is a gold-standard software widely used in the industry for its robust data analysis capabilities (Keizer et al., 2013, Nguyen et al., 2017). It was developed by Stuart Beal and Lewis Sheiner in the 1970 s (Keizer et al., 2013, Sheiner and Beal, 1980). Currently it is developed under ICON (https://www.iconplc.com). Another software in the 1970 s was NPEM (non-parametric expectation maximization algorithm) led by Dr. Roger Jelliffe and his team at the University of South California (Jelliffe et al., 2000). Currently, the NPEM software has been updated as an R package named “Pmetrics” (Neely et al., 2012). Both software’s were originally developed with the purpose of using them for drug dosing at the bedside(Keizer et al., 2018). During the 80’s, several other software’s were developed such as ADAPT and PopKinetics (Csajka and Verotta, 2006). In the decades that followed the release of these software’s, the field of population PK/PD modeling and simulation advanced and became an integral part of drug development (Csajka and Verotta, 2006, Holford, 1990, Keizer et al., 2018, Sheiner, 1969). Other software’s developed later include Monolix, PumasAI and Phoenix NLME. Monolix is part of the MonolixSuite and is designed for advanced pharmacometrics analysis (Lavielle and Mentré, 2007, Nguyen et al., 2017, Traynard et al., 2020). Developed by Lixoft, it offers a robust and user-friendly environment for model-based drug development, employing powerful algorithms for population PK/PD modeling. Monolix facilitates the handling of complex datasets and supports a wide range of statistical models, enabling investigators to perform NLME modeling with greater efficiency and precision. With its intuitive graphical user interface and extensive visualization tools, Monolix streamlines the model building and validation process, making it an indispensable tool in the pharmaceutical industry for optimizing dosing regimens, supporting regulatory submissions, and ultimately enhancing patient care. Additionally, Phoenix is a comprehensive software suite for advanced PK/PD modeling and simulation developed by Certara (Nguyen et al., 2017). It is designed to support the entire drug development process, from preclinical to clinical stages. Phoenix includes a range of tools, such as WinNonlin for non-compartmental analysis, NLME (Nonlinear Mixed Effects) for population modeling, and IVIVC (In Vitro-In Vivo Correlation) for establishing relationships between in vitro drug release and in vivo absorption. The platform is known for its scalability, flexibility, and integration with other software tools, making it a versatile choice for researchers and modelers in the pharmaceutical industry. Moreover, Pharmaceutical Modeling and Simulation (Pumas), which is developed by PumasAI, is a comprehensive platform for pharmaceutical modeling and simulation, providing a single tool for the entire drug development pipeline (Rackauckas et al., 2022). It is used for simulation and estimation of quantitative pre-clinical and clinical pharmacological models. It leverages the power of the Julia programming language to combine modern AI with traditional mechanistic models to gain massive computational efficiency.

When it comes to PBPK modeling, Gastroplus, PK-Sim, and Simcyp Simulators stand out as leading platforms, incorporating population-based demographics to predict pharmacokinetic outcomes in virtual patient populations(Agoram et al., 2001, Ezuruike et al., 2022, Romero et al., 2020, Silva et al., 2022, Willmann et al., 2003). These tools are particularly valued for their flexibility and community-driven development. Other noteworthy tools include MATLAB with its SimBiology toolbox, which allows for a more programming-oriented approach for modeling and simulation(Hosseini et al., 2020, Hosseini et al., 2018, Park and Kim, 2019, Vasić et al., 2024). Each of these software solutions offers a unique set of features, and the choice of tool often depends on the specific requirements of the modeling task at hand, including the complexity of the model, the nature of the data, regulatory considerations, and the user's familiarity with the software.

## 6 Examples of MID3 applications to inform decision-making

**Fig. 2:**
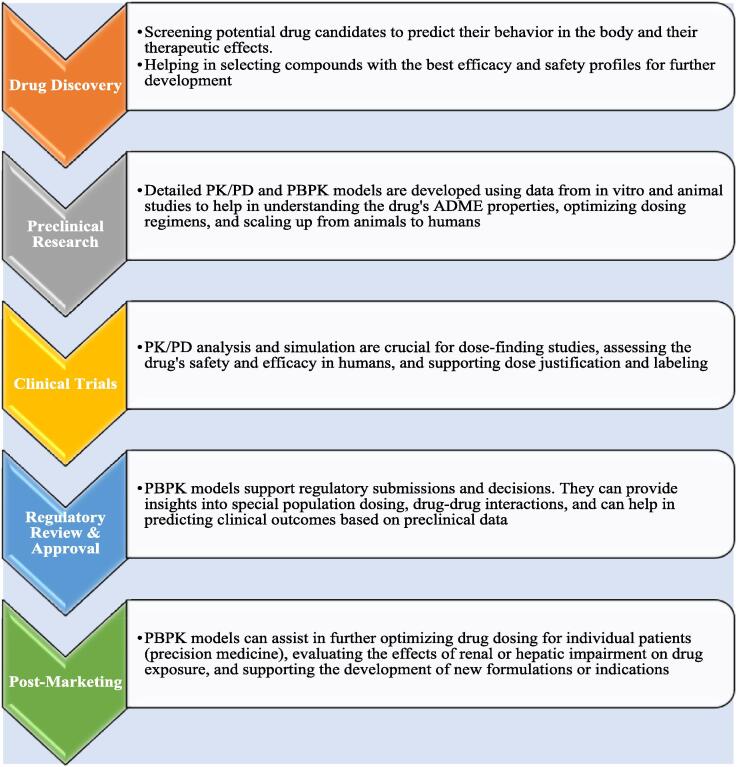
General applications of modeling and simulation in drug discovery and development.

MID3 applications span various stages, including target identification and validation, where computational models provide insights into disease mechanisms and potential drug targets, and predictive modeling forecasts the efficacy and safety of targeting specific pathways (Marshall et al., 2016). In Fig. 2, we summarized the applications of modeling and simulation across various stages of drug discovery and development.

**Fig. 3:**
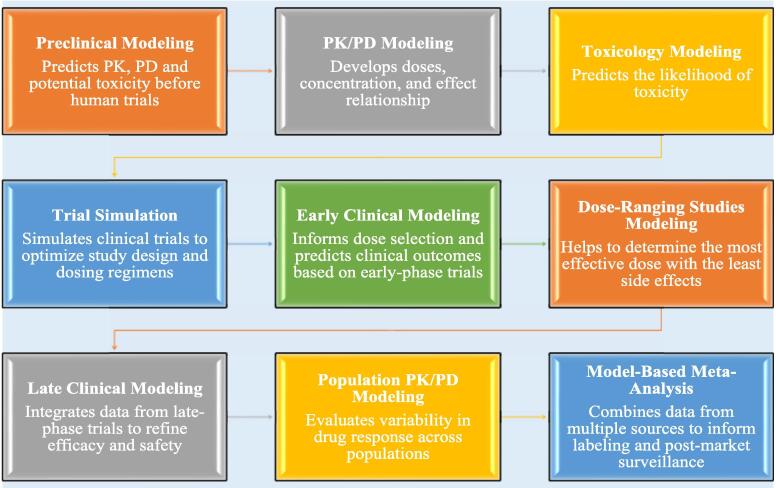
Types of modeling and simulation in different stages of the drug discovery and development processes. **Preclinical modeling** includes using of in vitro and in vivo studies and simulations to provide insights into drug behavior, efficacy, and safety prior to human trials. **PK/PD modeling** evaluates the relationship of PK data to PD effect (response). **Toxicology modeling** is used to predict toxicity, assess risk, and understand the mechanism of adverse effects based on exposure to chemical (e.g., dose–response model). **Trial simulation** helps in optimizing study design, dose, selection, and evaluating potential scenarios before actual trials are conducted. **Early clinical modeling** integrates preclinical and early-phase clinical trial results to predict drug behavior, help in dose selection, and the design of later-stage clinical trials. **Dose-ranging studies modeling** allows for exploring the relationship between different drug doses and their PK and PD effects to identify the optimal dose that maximizes efficacy while minimize toxicity. **Late clinical modeling** allows for integrating data from advanced clinical trials **(**phase III) to refine dose–response relationships, predict long term efficacy and, support regulatory decision making. **Population PK/PD modeling** analyzes drug concentration and response across diverse patient populations, accounting for variability in drug exposure and effects which help to identify factors influencing drug behavior, optimize dosing, and improve therapeutic outcomes for different subgroups. **Model-based *meta*-analysis** integrates data from multiple studies, allowing for comparison of drug efficacy and safety across studies which help to inform dose selection, predict clinical outcomes, and guide decision-making in drug development.

During lead optimization, MID3 guides the selection of compounds with optimal PK and PD profiles and aids in understanding dose–response relationships to select promising candidates (Marshall et al., 2016). In preclinical development, translational modeling predicts human responses based on animal data, while safety assessment models identify potential toxicity concerns. MID3 supports clinical trial design and execution through adaptive trials and optimal sample size determination, enhancing trial efficiency and reliability (Marshall et al., 2016). It facilitates regulatory interactions by providing robust modeling data for submissions and meeting regulatory requirements. Dose optimization and individualization are achieved by tailoring regimens based on patient characteristics and special populations, such as pediatrics and geriatrics (Marshall et al., 2016). In post-marketing, MID3 supports ongoing monitoring of drug safety and effectiveness through post-marketing surveillance models and continuous benefit-risk assessment. In Fig. 3, we highlighted some types of modeling and simulations that are used in drug discovery and development.

Overall, MID3 enhances the prediction of outcomes, optimizes trial designs, and informs strategic decisions, leading to more efficient and effective development of new therapies. In the following sections, we provided some examples of the applications of MID3 techniques.

### 6.1 Determining the FIH dose for transferring from preclinical to clinical phases

Estimation of the first-in-human (FIH) dose is of critical importance to earlier phases clinical trials (Zou et al., 2012). A well-established starting dose helps minimize the risk of adverse effects, ensures the well-being of study participants, facilitates the design of later-phase trials, helps allocate resources more effectively, and ultimately enables for a more efficient and streamlined development process. Methods for determining the FIH dose involve a combination of preclinical data, pharmacokinetic modeling, and safety considerations, and the most common approaches include allometric scaling, PK/PD, and PBPK(Jones et al., 2019, Jones et al., 2009, Miller et al., 2019). The primary objective of FIH trials is to identify a safe dose range for further investigation in drug development. The FDA and EMA guideline for the first in human dose provided recommendations for determining the initial dose of a new drug when it is first tested in humans (Table 1) (Shen et al., 2019). These guidelines emphasized the importance of conducting thorough preclinical studies to assess the safety and pharmacology of the drug before it is administered to humans. The initial dose should be carefully calculated based on factors such as the pharmacokinetics, pharmacodynamics, and toxicology of the drug.

PBPK models are widely used in drug discovery and development for predicting first in human dose(Miller et al., 2019). The appropriate starting dose for human trials can be estimated using PBPK modeling by integrating preclinical in-vitro and in-vivo data, considering factors like body weight, metabolic rates, gene expression, and other species-specific differences(Jones and Rowland-Yeo, 2013, Thiel et al., 2015). The availability of pharmacokinetic data from animal studies can be incorporated into the established model for qualification and optimization of the model's parameters. After qualification, the model can be extrapolated to predict human pharmacokinetics, accounting for physiological, biochemical, and genetic interspecies-difference.

PBPK modeling and simulation plays a critical role in selecting preclinical drug candidates for future drug development and guides the recommendation for the selection of the FIH dose. For example, when the COVID-19 crisis revealed the importance of an accelerated drug development process, a PBPK model was developed to predict the FIH dose for a monoclonal antibody (mAb) bamlanivimab (Chigutsa et al., 2022, Jones et al., 2019). Pharmacokinetic parameters of the mAb were characterized successfully based on data from preclinical studies. In combination with a SARS-COV2 viral dynamic model, PBPK model of bamlanivimab was used to predict the initial therapeutic dose that maintain lung tissue concentrations at or above the in-vivo IC_90_ over period of 4 weeks. Ultimately, bamlanivimab was approved for used in patients with COVID-19 under emergency use authorization in November 2020.

### 6.2 Guiding drug dosing in phase II/III clinical trials

During drug development, one of the main concerns is determining the appropriate dose for phase III or even phase II, in order to meet the pre-specified requirements for safety and efficacy endpoints. Population pharmacokinetics is an essential methodology for predicting PK characteristics and is critically required for drug approval. It offers several advantages over dose-titration studies in dose prediction, as it considers both between- and within-individual variability, as well as demographic characteristics and clinical disease states, including patients with renal/hepatic impairment (Lavé et al., 2016). Several studies have implemented modelling to guide dose selection for clinical trials(Landry et al., 2016, Wang et al., 2013). The robustness of the population pharmacokinetics model provides a solid evidence base for dose recommendations in later phases of drug development (i.e., phase II and phase III), utilizing drug exposure data from the previous phase (phase I) and expected clinical response.

Population pharmacokinetics analysis provides exposure information that is used as input for the exposure response relationship. By combining the estimated exposure (derived from the PK model) and the response (derived from the PD model), exposure response relationship can be predicted. This can then be used to guide dose selection for phase II/III trials using Monte Carlo simulation of varying dose regimens.

The rationale behind using both PK and PD to guide dose selection is that, in order to achieve the desired pre-specified safety and efficacy margins, one must first identify the target effect and use a PD model to predict the target concentration necessary to achieve the desired effect, and then use a PK model to predict the optimal dose required to achieve the target concentration. This approach considers variability in the PK-PD target and effect site exposures in dose prediction (Chien et al., 2005, Derendorf and Meibohm, 1999). Incorporating clinical data allows for the prediction and confirmation of responses in patient subgroups that may be at increased risk for adverse events or decreased efficacy.

### 6.3 Informing drug label for special populations

In the context of clinical pharmacology, the term “special population” refers to specific groups of individuals who may have unique characteristics or factors that can affect their response to medications (Grimsrud et al., 2015). This can include groups such as pediatrics, elderly individuals, pregnant women, obese, individuals with certain medical conditions such as renal and hepatic impairment, or those taking multiple medications (Marshall et al., 2016). These patients have unique physiological characteristics and drug response profiles compared to adult healthy populations, making it challenging to determine the appropriate dosing and safety profiles for these populations(Anderson and Holford, 2013, Costantine, 2014, Verbeeck, 2008, Verbeeck and Musuamba, 2009). Population PK/PD, as well as PBPK modeling, play crucial roles in drug development in these population where physiological differences from healthy adults significantly affect how drugs are absorbed, distributed, metabolized, and excreted(Han et al., 2021, Heimbach et al., 2021, Malik et al., 2020, Vinks et al., 2015). They help to tailor drug therapy to the unique physiological needs, ensuring that medications are used safely and effectively in these vulnerable populations. These tools also facilitate the rational design of clinical trials and support regulatory decision-making, ultimately improving access to therapeutics.

As examples, we report some of MIDD applications for special populations in relation to anticoagulants. A population pharmacokinetic model for a low molecular weight heparin (dalteparin) was constructed based on a clinical study involving 98 children to recommend starting doses for different pediatric groups (Damle et al., 2021). The clinical observations were best described by two-compartment model using NONMEM software. The analysis suggested that the starting doses of dalteparin administered subcutaneously for pediatrics ages 1 month to 2 years, 2 years to 8 years, and 8 years to 19 years should be 150 IU/kg twice daily, 125 IU/kg twice daily, and 100 IU/kg twice daily, respectively. Results from this study contributed to the approval of dalteparin by the FDA for venous thromboembolism in different pediatrics groups. Terrier et al (2022) reported several population pharmacokinetic models used for dosing optimization of anticoagulants(Terrier et al., 2022). Similarly, PBPK models are widely used and accepted in individualizing drug dosing in special populations. For instance, PBPK models for apixaban and rivaroxaban were constructed to guide dosage regimen in pediatrics, geriatrics, patient taking multiple drugs, and in patients with various degree of renal and hepatic dysfunction(Wen et al., 2022, Willmann et al., 2021, Xu et al., 2021, Xu et al., 2024). The models were able to accurately predict the PK of the drugs when taken alone and when taken concomitantly with other drugs. In patients with varying degrees of kidney and liver impairment, the PBPK models correctly estimated the increase in drug exposure for each class. For pediatrics, PBPK modeling and simulation found that the influence of renal impairment on drug exposure might be greater in infant less than one year compared to older children (Xu et al., 2021).

### 6.4 Supporting drug development in rare diseases

The term “rare disease” is often defined differently depending on the context and the legislation of different countries. In the context of the United States, the Orphan Drug Act of 1983 is a key piece of legislation that provides a definition for rare diseases and conditions, primarily for the purpose of encouraging the development and commercialization of drugs for the treatment of these diseases. According to the Orphan Drug Act, a rare disease or condition is one that affects fewer than 200,000 people in the United States(Aronson, 2006, Wellman-Labadie and Zhou, 2010). The Act provides incentives for pharmaceutical companies to develop treatments for rare diseases, which are often referred to as “orphan drugs” because without these incentives, the small market would not typically justify the investment required for drug development. Drug development for rare diseases faces several challenges, including lack of knowledge on the natural disease progression, disease heterogeneity, and the small number of eligible patients for randomized controlled trials.

As an example of the successful application of the MID3 to support development of drugs for rare diseases, population pharmacokinetic simulations were performed to predict adalimumab exposure in adolescent hidradenitis suppurativa patients without additional clinical PK data(Bi et al., 2019). Based on these simulations, dosing based solely on body weight resulted in inadequate drug exposure, and the study recommended a weight-tiered dosing regimen for adolescent HS patients to achieve similar drug exposure as adult hidradenitis suppurativa patients across the weight range. Consequently, based on these findings, FDA recommended a weight-tiered dosing regimen for adalimumab in adolescent hidradenitis suppurativa patients.

Furthermore, optimizing the selection of patients in clinical trials is critical to effectively obtain the necessary information on drug safety and efficacy. Disease progression models can serve as a valuable tool to guide patient selection strategies. This is demonstrated in the case of childhood-onset dystrophinopathy(Haber et al., 2021). Disease progression modelling was used to predict the impact of genetic variant and loss of ambulation(Li et al., 2022). The model predicted that subjects with specific genetic variants had a lower risk of loss of ambulation. These results suggest that genotyping may help guide patient selection in clinical trials. These studies highlighted the importance of MID3 in accelerating drug development, particularly in the treatment of rare diseases.

## 7 MID3 considerations for MENA region

The MENA region faces a unique set of health challenges, specific to populations demographics, genetic background, and health and socioeconomic status (Akala and El-Saharty, 2006, Alsultan et al., 2023, Katoue et al., 2022, Nair et al., 2013, Wakil et al., 2015). Developing drugs tailored to these needs can significantly improve public health outcomes. Investing in drug development can boost the pharmaceutical sector, creating jobs, fostering innovation, and contributing to economic diversification. Local drug development can reduce reliance on imported medications, ensuring more stable and affordable access to essential medicines. This is crucial during global supply chain disruptions, such as those seen during the COVID-19 pandemic (Ibrahim et al., 2020). Strengthening drug development capabilities promotes scientific research and innovation. It also helps build local expertise and infrastructure, making the region a hub for pharmaceutical research. Adopting MID3 approach in the MENA region could significantly enhance the efficiency and effectiveness of the pharmaceutical development process. MID3 can help develop personalized treatment plans based on genetic, demographic, and disease-specific data. This is particularly relevant in the MENA region, where the populations have certain genetic and physiological characteristics. Regulatory bodies can use MID3 to make more informed decisions regarding drug approvals, ensuring that new medications are both effective and safe for the MENA populations. MID3 allows for better allocation of resources by identifying promising drug candidates early and discontinuing those with low potential. This optimizes the use of financial and human resources in drug development projects.

It is very important to create a virtual population specific to MENA ethnicity for PBPK modeling to simulate PK profile more precisely. To develop a virtual human individual representing a MENA population in a PBPK platform, comprehensive demographic, physiological, biochemical, and clinical data are essential. Demographic information should include mean body weight, height, and BMI distribution. Physiological data must cover organ sizes, blood flow rates, and tissue compositions. Biochemical data should encompass data such as enzyme activity levels. Additionally, clinical data from studies conducted on the MENA populations, including PK profiles and PD, are necessary. In Table 3, we highlighted some key factors in order to successfully implement the MID3 approaches specific for MENA region.

**Table 3:** Key factors to implement MID3 in the MENA region.

**Factor**	**Definition**
Regulatory Framework	• Establish clear guidelines and regulatory frameworks that incorporate MID3 principles, and regulatory bodies need to recognize and accept model-based evidence in drug approval processes. Provide training for regulatory personnel to understand and evaluate MIDD approaches, ensuring they can effectively assess model-based submissions.
Infrastructure	• Invest in high-performance computing infrastructure and advanced software tools necessary for complex modeling and simulation tasks. Develop robust data management systems to handle large volumes of data required for MID3, ensuring data integrity and accessibility.
Skilled Workforce	• Establish academic and training programs focused on pharmacometrics, biostatistics, computational biology, and related fields to develop a skilled workforce. Partner with international institutions and experts to provide specialized training and knowledge transfer in MID3 techniques.
Collaboration and Partnerships	• Foster partnerships between pharmaceutical companies, academic institutions, and research organizations to share knowledge, resources, and expertise. Engage with global organizations and regulatory bodies to stay updated on best practices, standards, and technological advancements in MID3.
Research and Development (R&D) Investment	• Increase investment in R&D specifically for MID3 projects, encouraging innovation and development of new modeling techniques and applications. Provide grants and other financial support to companies and institutions that invest in MID3-related research.
Data Availability and Sharing	• Create comprehensive health databases with high-quality, real-world data that can be used for modeling and simulation. Develop policies that facilitate data sharing while ensuring patient privacy and data security, promoting collaborative research.
Public Awareness and Support	• Raising awareness about the benefits of MID3 among healthcare professionals, policymakers, and the public. Encourage pharmaceutical companies and research institutions to adopt a culture that values and integrates MID3 into their development processes.
Pilot Projects and Case Studies	• Initiate pilot projects to demonstrate the effectiveness and benefits of MID3 in real-world drug development scenarios. Document and share successful case studies to build confidence and illustrate the practical advantages of MID3.
Policy and Government Support	• Promoting and supporting the adoption of MID3 through policies, funding, and strategic initiatives. Encourage public–private partnerships to leverage resources and expertise from both sectors in advancing MID3.

## 8 Educational resources

Barrett et al (2008) advocated for structured education and training programs to equip scientists with the necessary skills to excel in the pharmacometrics field(Barrett et al., 2008). They listed academic sites/universities offering curriculum related to pharmacometrics and some centers and web sites providing training programs. They also highlighted multidisciplinary nature of the pharmacometrics requiring expertise in biology, pharmacology and quantitative sciences. Schmidt et al (2023) discussed the comprehensive framework needed to effectively train future pharmacometricians (Schmidt et al., 2023). They outlined several key requirements for training programs, emphasizing the need for a strong foundation in quantitative and system pharmacology and the integration of advanced computational tools. Additionally, there is a call for more collaborative efforts between academia, industry, and regulatory bodies to enhance training quality and relevance. They also advocated for the development of global training networks to facilitate knowledge exchange and foster a diverse and inclusive workforce in pharmacometrics. Expectations for trainees include proficiency in MID3 and continuous professional development to keep pace with the rapidly evolving field.

Moreover, ACCP journal published a collection of educational papers on PBPK modeling and simulation which can be very helpful resources (Chaudhury et al., 2020, Franchetti and Nolin, 2020, Li et al., 2020, Malik et al., 2020, Rostami-Hodjegan and Toon, 2020, Samineni et al., 2020, Sharma et al., 2020). On the other hand, tutorials for using NONMEM for population PK/PD modeling and simulation have been published in more details(Bauer, 2019a, Bauer, 2019b, Bauer et al., 2021, Keizer et al., 2013). We have listed some additional helpful educational resources in Table 4.

**Table 4:** Some educational resources for MID3.

**Sources**	**Description**
Textbooks	• Applied Pharmacometrics by Stephan Schmidt & Hartmut Derendorf Rowland and Tozer's Clinical Pharmacokinetics and Pharmacodynamics: Concepts and Applications Pharmacokinetic and Pharmacodynamic Data Analysis: Concepts and Applications by Johan Gabrielson and Daniel Weiner Applied Clinical Pharmacokinetics Shargel & Yu's Applied Biopharmaceutics & Pharmacokinetics Physiologically Based Pharmacokinetic (PBPK) Modelling and Simulations: Principles, Methods, and Applications in the Pharma Industry by Sheila Annie Peters Introduction to Population Pharmacokinetic / Pharmacodynamic Analysis with Nonlinear Mixed Effects Models by Joel S. Owen and Jill Fiedler-Kelly Pharmacokinetic-Pharmacodynamic Modeling and Simulation by Peter L. Bonate Introduction to Pharmacokinetics and Pharmacodynamics: The Quantitative Basis of Drug Therapy by Thomas N. Tozer, Malcolm Rowland Pharmacometrics: The Science of Quantitative Pharmacology 1st Edition by Ene I. Ette, Paul J. Williams The Art and Science of Physiologically-Based Pharmacokinetics Modeling by Rodrigo Cristofoletti and Amin Rostami-Hodjegan (New PBPK modeling textbook will be released in July 2024)
Online Courses and Workshops	• Buffalo Pharmacometrics Workshops(https://pharmacy.buffalo.edu/pkpd-workshops) Icon workshops (https://www.iconplc.com/news-events/events/workshops) Certara university https://www.certarauniversity.com/store https://lixoft.com/lixoft-university/ https://www.simulations-plus.com/software/slp-university-program/ https://metrumrg.com/courses/
Scientific Journals	• Clinical Pharmacokinetics Pharmacometrics & Systems Pharmacology (PSP) Clinical Pharmacology & Therapeutics (CPT) Clinical and Translational Science (CTS) The Journal of Clinical Pharmacology Clinical Pharmacology in Drug Development Pharmaceutics European Journal of Drug Metabolism and Pharmacokinetics Drug Metabolism and Pharmacokinetics Journal of Pharmacokinetics and Pharmacodynamics British Journal of Clinical Pharmacology
Professional meeting and societies	• American Conference of Pharmacometrics (ACOP) Population Approach Group Europe (PAGE) Population Approach Group in Australia and New Zealand (PAGANZ) Asian Pharmacometrics Network (APN) World Conference on Pharmacometrics (WCOP) American Association of Pharmaceutical Scientists (AAPS) American College of Clinical Pharmacology (ACCP) American Society for Clinical Pharmacology and Therapeutics (ASCPT) International society of pharmacometrics Pharmacometrics Africa
Regulatory Guidance Documents	• https://www.federalregister.gov/documents/2018/04/17/2018–08010/pilot-meetings-program-for-model-informed-drug-development-approaches
Software Tools	**Monolix:** https://monolix.lixoft.com/wp-content/uploads/sites/17/2019/10/maryland_CS1-1.pdf https://monolix.lixoft.com/wp-content/uploads/sites/17/2019/10/maryland_CS2-1.pdf https://monolix.lixoft.com **NONMEM**: https://www.iconplc.com/solutions/technologies/nonmem **Phoenix**: https://www.certara.com/software/phoenix-nlme/ **Simcyp** https://www.certara.com/software/pbpk-modeling-and-simulation/ **PK-Sim:** https://www.open-systems-pharmacology.org https://github.com/Open-Systems-Pharmacology/OSP-based-publications-and-content/labels/Tutorial **Pumas:** **https://pumas.ai/our-products/products-suite/pumas** **GastroPlus** https://www.simulations-plus.com/software/gastroplus/ **SimBiology:** https://www.mathworks.com/products/simbiology.html **PhysPK:** https://www.physpk.com
Industry Conferences and Workshops	• Population Approach Group in Europe (PAGE) American Conference on Pharmacometrics (ACoP)
Tutorials	• Basic Concepts in Physiologically Based Pharmacokinetic Modeling in Drug Discovery and Development(Jones and Rowland-Yeo, 2013) Basic concepts in population modeling, simulation, and model-based drug development(Mould and Upton, 2012) Basic concepts in population modeling, simulation, and model-based drug development − Part 2: Introduction to pharmacokinetic modeling methods(Mould and Upton, 2013) Basic concepts in population modeling, simulation, and model-based drug development: Part 3-introduction to pharmacodynamic modeling methods(Upton and Mould, 2014) Tutorial for $DESIGN in NONMEM: Clinical trial evaluation and optimization(Bauer et al., 2021) NONMEM Tutorial Part II: Estimation Methods and Advanced Examples(Bauer, 2019a) Modeling and simulation workbench for NONMEM: Tutorial on Pirana, PsN, and Xpose (Keizer et al., 2013) NONMEM Tutorial Part I: Description of Commands and Options, With Simple Examples of Population Analysis(Bauer, 2019b) Applied Concepts in PBPK Modeling: How to Build a PBPK/PD Model (Kuepfer et al., 2016) https://tutorials.pumas.ai Applied concepts in PBPK modeling: how to extend an open systems pharmacology model to the special population of pregnant women(Dallmann et al., 2018) Guide to development of compound files for PBPK modeling in the Simcyp population-based simulator(Ezuruike et al., 2022) A Time to Event Tutorial for Pharmacometricians(Holford, 2013) A Tutorial on Target-Mediated Drug Disposition (TMDD) Models(Dua et al., 2015) Open Source Pharmacokinetic/Pharmacodynamic Framework: Tutorial on the BioGears Engine(McDaniel et al., 2019) A Tutorial on RxODE: Simulating Differential Equation Pharmacometric Models in R (Wang et al., 2016) Physiologically-Based Pharmacokinetic Modeling for Drug Dosing in Pediatric Patients: A Tutorial for a Pragmatic Approach in Clinical Care(van der Heijden et al., 2023) Quantitative Systems Pharmacology and Physiologically- Based Pharmacokinetic Modeling With mrgsolve: A Hands-On Tutorial (Elmokadem et al., 2019) Tutorial on model selection and validation of model input into precision dosing software for model-informed precision dosing(Taylor et al., 2023) Physiologically Based Pharmacokinetic Modeling and Simulation in Pediatric Drug Development(Maharaj and Edginton, 2014) Establishing Good Practices for Exposure–Response Analysis of Clinical Endpoints in Drug Development (Overgaard et al., 2015) ATLAS mPBPK: A MATLAB-Based Tool for Modeling and Simulation of Minimal Physiologically-Based Pharmacokinetic Models(Mavroudis et al., 2019) Model Evaluation of Continuous Data Pharmacometric Models: Metrics and Graphics (Nguyen et al., 2017) Modeling and simulation of count data (Plan, 2014) Model-Based Discovery and Development of Biopharmaceuticals: A Case Study of Mavrilimumab (Wang et al., 2018) Use of Modeling and Simulation in the Design and Conduct of Pediatric Clinical Trials and the Optimization of Individualized Dosing Regimen(Stockmann et al., 2015) A model qualification method for mechanistic physiological QSP models to support model-informed drug development (Friedrich, 2016) Establishing Best Practices and Guidance in Population Modeling: An Experience With an Internal Population Pharmacokinetic Analysis Guidance(Byon et al., 2013) The Many Flavors of Model-Based Meta-Analysis: Part I—Introduction and Landmark Data (Boucher and Bennetts, 2016) Many Flavors of Model-Based Meta-Analysis: Part II – Modeling Summary Level Longitudinal Responses (Boucher and Bennetts, 2018)

## 9 Conclusion

The integration of MID3 presents a unique and significant opportunity for the MENA region to advance its pharmaceutical research and healthcare outcomes. This review highlights the transformative potential of MID3 to address regional health challenges through the use of sophisticated quantitative models such as population PK/PD and PBPK modeling. To fully realize the potential of MID3, it is imperative for MENA countries to invest in developing a skilled workforce adept at utilizing these advanced methodologies. Harmonizing regulatory frameworks to support model-based approaches and fostering collaborative research environments are also critical steps. By prioritizing the integration of MID3, MENA nations can not only enhance their own healthcare systems but also contribute to global advancements in pharmaceutical research. In conclusion, the adoption of MID3 in the MENA is not just an opportunity but a necessity. Policymakers, academic institutions, and industry stakeholders must work together to create a supportive ecosystem that encourages innovation and collaboration.

## CRediT authorship contribution statement

**Mohammed S. Alasmari:** Writing – review & editing, Writing – original draft, Conceptualization. **Salwa Albusaysi:** Writing – review & editing, Writing – original draft, Conceptualization. **Marwa Elhefnawy:** Writing – review & editing, Writing – original draft, Conceptualization. **Ali M. Ali:** Writing – review & editing, Writing – original draft, Conceptualization. **Khalid Altigani:** Writing – review & editing, Writing – original draft. **Mohammed Almoslem:** Writing – review & editing, Writing – original draft. **Mohammed Alharbi:** Writing – review & editing, Writing – original draft. **Jahad Alghamdi:** Writing – review & editing, Writing – original draft, Conceptualization. **Abdullah Alsultan:** Writing – review & editing, Writing – original draft, Conceptualization.

## Declaration of Competing Interest

The authors declare that they have no known competing financial interests or personal relationships that could have appeared to influence the work reported in this paper.
